# Non-wounding contact-based Inoculation of fruits with fungal pathogens in postharvest

**DOI:** 10.1186/s13007-024-01214-2

**Published:** 2024-06-02

**Authors:** Adrian O. Sbodio, Saskia D. Mesquida-Pesci, Nancy Yip, Isabela Alvarez-Rojo, Elia Gutierrez-Baeza, Samantha Tay, Pedro Bello, Luxin Wang, Barbara Blanco-Ulate

**Affiliations:** 1 Department of Plant Sciences, University of California, Davis, CA 95616 USA; 2 Department of Food Science, University of California, Davis, CA 95616 USA

**Keywords:** Fruit diseases, Mold, Penicillium, Botrytis, Orange, Tomato, Apple, Early detection

## Abstract

**Background:**

Fungal pathogens significantly impact the quality of fruits and vegetables at different stages of the supply chain, leading to substantial food losses. Understanding how these persistent fungal infections occur and progress in postharvest conditions is essential to developing effective control strategies.

**Results:**

In this study, we developed a reliable and consistent inoculation protocol to simulate disease spread from infected fruits to adjacent healthy fruits during postharvest storage. We tested different combinations of relevant fruit commodities, including oranges, tomatoes, and apples, against impactful postharvest pathogens such as *Penicillium digitatum*, *Penicillium italicum, Botrytis cinerea*, and *Penicillium expansum.* We assessed the efficacy of this protocol using fruits treated with various postharvest methods and multiple isolates for each pathogen. We optimized the source of infected tissue and incubation conditions for each fruit-pathogen combination. Disease incidence and severity were quantitatively evaluated to study infection success and progression. At the final evaluation point, 80% or higher disease incidence rates were observed in all trials except for the fungicide-treated oranges inoculated with fungicide-susceptible *Penicillium spp.* isolates. Although disease incidence was lower in that particular scenario, it is noteworthy that the pathogen was still able to establish itself under unfavorable conditions, indicating the robustness of our methodology. Finally, we used multispectral imaging to detect early *P. digitatum* infections in oranges before the disease became visible to the naked eye but after the pathogen was established.

**Conclusions:**

We developed a non-invasive inoculation strategy that can be used to recreate infections caused by contact or nesting in postharvest. The observed high disease incidence and severity values across fruit commodities and fungal pathogens demonstrate the robustness, efficacy, and reproducibility of the developed methodology. The protocol has the potential to be tailored for other pathosystems. Additionally, this approach can facilitate the study of fruit-pathogen interactions and the assessment of innovative control strategies.

## Background

Nearly one-third of the world’s food production is lost or wasted through the food supply chain, according to the Food and Agriculture Organization (FAO) [1]. Fungal pathogens are responsible for causing diseases like rots and molds that result in reduced product quality, shelf-life, and market value, leading to significant losses of harvested fruits and vegetables in postharvest [2, 3].

Fungal pathogens can gain access to the fruit tissues in different ways: by establishing latent infections of flowers, exploiting wounds and natural openings (e.g., pedicels, lenticels, and stem ends), or by directly penetrating the host cuticle [4]. Vector insects can also cause damage, which is a common entry point for pathogens [5]. Additionally, mishandling and physical damage of the products during harvest, sorting, packing, transportation, cold storage, and retailing contribute to a higher incidence of disease.

The germination of fungal spores requires moisture and stimulation from host solutes that diffuse into the initial penetration site [6]. After germination, specific signals from the plant surface trigger the formation of specialized fungal structures that enable the pathogen to penetrate the plant cell walls and establish infection. Once the disease is well established in one fruit, it spreads quickly to adjacent healthy fruit, a process known as nesting. A well-known nesting pathogen is *Botrytis cinerea*, which releases airborne conidia that readily nest on damaged or senescent fruits, initiating decay and facilitating further spread [7–9]. *Rhizopus stolonifer*, the causal agent of Rhizopus rot in various fruits and vegetables, is another prominent example of a nesting pathogen. Following spore germination, *R. stolonifer* produces mycelial stolons that attach to the host surface, enabling it to colonize healthy fruits and initiate infections [10]. *Penicillium spp.* are commonly considered wound-dependent pathogens [9, 11]; however, it is commonly observed that if the fungi are initially established in a rich food source like a decaying fruit, the mycelium can readily invade the tissues of an adjacent healthy fruit. This phenomenon is how an initial low incidence of green or blue mold in a packinghouse storage facility can develop into major losses after prolonged fruit storage [9, 12, 13].

Integrated pest management strategies have been developed to reduce or eliminate fruit infections, including synthetic fungicides before and after harvest, biological control agents, essential oils, cold storage, and modified atmosphere packaging [14]. To test the effectiveness of these strategies, reliable laboratory or field-based inoculation methods are required to obtain quantitative data that goes beyond subjective ordinal rating scales that may be influenced by human bias. While natural infections can provide insights into disease dynamics in real-world scenarios, they can be unpredictable and impacted by environmental factors, making it difficult to control and replicate experimental conditions. Therefore, the study of plant-pathogen interactions generally relies on pathogen inoculation techniques.

Dip and spray are two common inoculation methods where fruits are covered in a fungal spore suspension by submersion or application with an atomizer. These methods allow for uniform, whole-fruit inoculation but may result in lower disease incidence and severity due to the challenges in standardizing the process. Another method is wound inoculation, which involves creating artificial entry points on the fruit surface before applying the fungal spore suspension or mycelial plug. This method simulates wounds and enables precise and reproducible experiments. Still, it may not accurately represent the natural infection process as it bypasses the initial steps of adhesion and penetration on the plant tissues. As of present, our comprehensive literature review has not revealed any reported methods that faithfully replicate the postharvest nesting phenomenon.

This study presents a novel methodology for assessing postharvest infections of persistent fungal pathogens through contact-based inoculation of fruits. We optimized this protocol using four impactful fungal pathogens and postharvest commodities that are commonly affected by fungal disease: *Botrytis cinerea* and *Penicillium expansum* in tomato and apple, respectively, and *Penicillium italicum* and *Penicillium digitatum* in orange. *B. cinerea* is the causal agent of gray mold, a devastating disease that causes billion-dollar losses on fruit commodities worldwide [15–19]. *P. italicum* and *P. expansum*, causal agents of blue mold, and *P. digitatum*, causal agent of green mold, are significant postharvest diseases [11, 20, 21]. Our protocol unveils new possibilities for testing disease management strategies and studying the nesting behavior of postharvest fungal pathogens.

## Methods

**Fig. 1:**
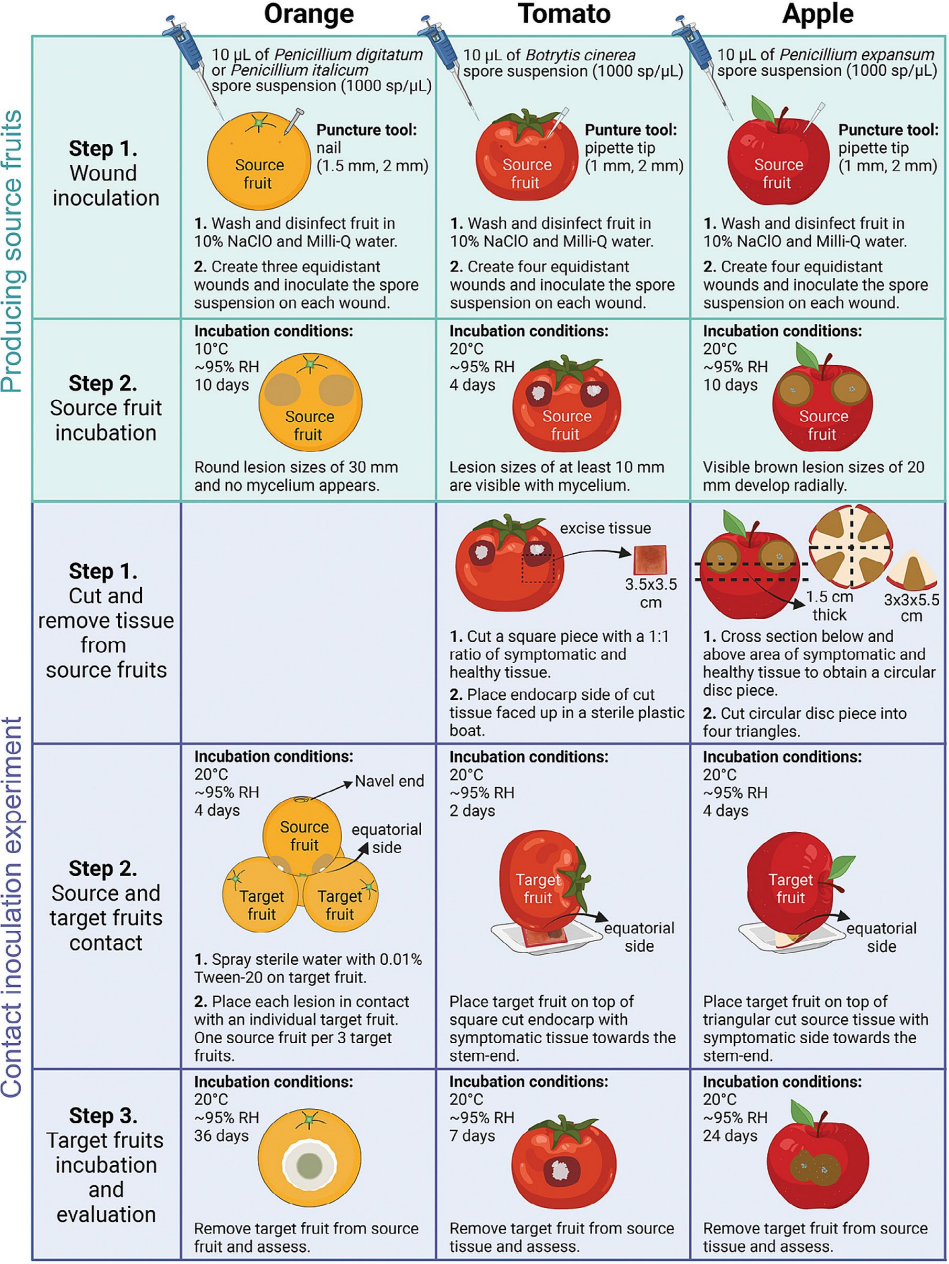
Non-wounding contact inoculation method of fungal pathogens in fruit. The protocol consists of two major steps: producing the source fruits and setting up the contact-inoculation experiment. First, fruits are wound-inoculated on the stem end using a 1,000 conidia/µL suspension and incubated under high relative humidity until lesion sizes are visible. Whole fruits with active lesions were used as the source inoculum for oranges, whereas tissue sections containing symptomatic and asymptomatic tissues were used instead for tomatoes and apples. Source fruits/tissues and target fruits were placed in contact and incubated at high relative humidity. After the fungal infections were successfully established in the target fruit, the source was removed, and the target was incubated under high relative humidity for further assessment

We aimed to find a reliable and effective way of inducing fungal infections by direct contact of infected fruits or tissues (source) with healthy fruits (target). Figure 1 provides a step-by-step visual workflow of the contact inoculation protocol, and details of the materials and methodology can be found in subsequent sections.

### Plant material: source and target fruits

Navel oranges (*Citrus sinensis* Osbeck), untreated and treated with the fungicide Imazalil (IMZ), were obtained from a packinghouse in Bakersfield, California. Commercial oranges treated with a fungicide mix of TBZ, IMZ, and fludioxonil and coated with wax were purchased from a local retail market. Tomatoes (*Solanum lycopersicum*) cvs. ‘Celebrity,’ ‘Shady lady,’ and ‘Rutgers’ were grown under standard field conditions in the Department of Plant Sciences Field Facilities at the University of California, Davis, during the 2021 season. Red ripe (RR) fruits were harvested 42 days post-anthesis (dpa). No fungicide or wax treatments were applied to field-grown tomatoes. Additionally, Village Farms International Inc. ‘Beefsteak’ tomatoes grown under commercial greenhouse conditions and using conventional practices (i.e., fungicide applications during production and postharvest) were purchased from a local retail market. Apples (*Malus domestica*) cv. ‘Gala’ commercially grown in Washington, were treated with pyrimethanil, fludioxonil, thiabendazole (TBZ), and ethoxyquin. Apples were coated with vegetable and/or shellac-based wax.

### Fungal isolates

Fungal isolates displaying fungicide sensitivity (wild-type, WT) or fungicide resistance (FR) were provided by Prof. James E. Adaskaveg (University of California, Riverside). All isolates were obtained from packing houses throughout the state of California (USA). The isolates corresponded to *B. cinerea* (FR4635), *P. expansum* (FR3400), and *P. digitatum* (FR3189), in addition to *P. italicum* WT (WT3212) and *P. digitatum* WT (WT2388) (Table 1).

**Table 1:** List of fungal isolates used in this study

Fungal Species	Host Species	Isolation Year	Fungicide Resistance Phenotype
*B. cinerea* B05.10	Tomato (*Solanum lycopersicum*)	n/a	Sensitive to all postharvest fungicides
*B. cinerea* FR4635	Apple (*Malus domestica*)	2011	Resistant to Fludioxonil, Thiabendazole, and Pyrimethanil
*P. expansum* FR3400	Lemon (*Citrus limon*)	2008	Resistant to Pyrimethanil
*P. digitatum* WT2388	Lemon (*Citrus limon*)	2001	Sensitive to all postharvest fungicides
*P. digitatum* FR3189	Lemon (*Citrus limon*)	2003	Resistant to Imazalil
*P. italicum* WT3212	Lemon (*Citrus limon*)	2003	Imazalil-sensitive

*B. cinerea* FR4635 was isolated from apple (*Malus domestica*) blossom tissues in Fall 2011 and displayed resistance against fludioxonil, thiabendazole (TBZ) and pyrimethanil. *P. expansum* FR3400 was isolated from lemon (*Citrus limon*) fruits sampled in a packinghouse in Bakersfield, California, in 2008 and displayed resistance against pyrimethanil. *P. digitatum* FR3189, was also isolated from lemon fruits sampled in a packinghouse in Bakersfield, California, in May 2003. Although sensitive to fludioxonil and azoxystrobin, *P. digitatum* FR3189 is moderately resistant to imazalil (IMZ) and highly resistant to TBZ.

Fungal isolates were grown on potato dextrose agar (PDA; Difco Laboratories, Detroit, MI, US) at room temperature until sporulation (7–10 days). Conidia were harvested from sporulating cultures by washing agar surface in sterile water with 0.01% (vw/v) Tween-20 followed by filtering to remove hyphal fragments through a double layer cotton cloth. For all fungal isolates, conidia suspension was quantified in a haemocytometer and diluted to 1000 conidia/µL. Fungicide resistance phenotypes were confirmed using the spiral gradient dilution method [22].

### Source fruit preparation

We conducted several trials to determine the best source of fruit tissues for contact-inoculation. The developed inoculation method involves (1) producing the source fruits through wound inoculation, and (2) preparing the non-wounded target fruits. The selected source and target fruits ideally should not have any surface imperfections such as scars, wounds, or bruises, and apples should have an intact pedicel. Fruits were first disinfected in 10% sodium hypochlorite, rinsed twice in sterile Milli-Q, and dried with sterile tissue paper (e.g., Kimwipes™, Kimberly-Clark, US) before inoculation.

Source fruits were wounded multiple times on the stem end. For oranges, three equidistant wounds were created using a sterile nail (1.5 mm wide, 2 mm deep) following the protocol developed by Vilanova et al. [23]. For tomatoes and apples, equidistant wounds were created in four locations with a sterile pipette tip (1 mm wide, 2 mm deep). Each wound was inoculated with 10 µL of a fungal spore suspension (1000 conidia/µL). Oranges were inoculated with *P. italicum* or *P. digitatum*, while tomatoes and apples were inoculated with *B. cinerea* and *P. expansum*, respectively. Source fruits were incubated under high relative humidity (90–95%) at 10 °C for 10 (mycelium absent) and 13 (mycelium visible) days for orange, 20 °C for 4 days for tomato, and 10 days for apple. By then, the source fruits should have developed lesion sizes of about 30, 10, and 20 mm for oranges, tomatoes, and apples, respectively.

In initial attempts, we used culture media plugs with well-established fungal mycelial growth as the inoculum source. However, this method was not always successful and did not accurately mimic how fungal infections occur while handling and storing fresh produce.

### Selection of inoculum: whole fruits and fruit tissue sections

Two different inoculum sources were explored for the contact inoculation: whole fruits and fruit tissue sections. When using whole fruits as the source, target fruits were placed on a plastic boat lying on the equatorial region. Source fruits were then placed on top of the target fruits so that the inoculated stem end region would be in contact with the equatorial region of the target. Using the whole fruits was the most effective way to contact-inoculate oranges. Source fruits with visible mycelium and with visibly macerated tissue radially spread from the initial wound but no mycelium were placed on top of the equatorial side of three target oranges, with each infection site of the source orange in direct contact with one of the target oranges. The contact point between the source and target oranges was pre-wetted by spraying sterile water containing 0.01% (v/v) Tween-20. Successful infections occurred when using oranges that did not have visible mycelium, while those with external mycelium failed to infect the target fruit.

For tomatoes and apples, the contact inoculation procedure failed when using whole fruits due to leakage and accumulation of juices from the source fruits during incubation, leading to off-target infections in non-contact points. We then decided to use tissue sections instead of whole fruits to ensure a successful and uniform inoculation process. For tomatoes, a 3.5 × 3.5 cm, square-shaped pericarp section containing healthy/asymptomatic and symptomatic tissue in a 1:1 ratio was used. Similarly, for apples, a cross-cut 1.5 cm thick was performed below and above the limit between symptomatic and asymptomatic tissue. The resulting disc was then cut into four 3 × 3 × 5.5 cm triangles containing decayed and macerated tissue in the middle and asymptomatic tissue on the sides. Source tissue sections of tomato and apple were placed on plastic boats, with the endocarp facing upwards for the tomato. Individual target tomatoes and apples with the equator side as the contact point were placed on top of their respective source tissue sections.

### Target fruit inoculation, incubation, and evaluation

The contact inoculation time was determined as the minimum time needed for successful disease development in the target fruits once the source fruits were detached. This corresponded to two days for tomatoes and four days for treated apples and oranges (fungicide and wax). For control oranges (untreated with fungicide or wax) and fungicide-treated oranges (without wax), the contact time was reduced to two days when using *P. italicum* WT and 1.5 days when using *P. digitatum* WT due to their advanced infection rates and aggressiveness on control and fungicide treated oranges. In all cases, contact inoculation was performed at room temperature, and target and source fruits were stored in high-humidity chambers (90–95%).

Following the contact inoculation, the source whole fruits or tissue sections were removed, and target fruits were stored at 20 °C under high relative humidity (90–95%) until mycelium reached the equatorial region, or until evaluations were completed. Target oranges inoculated with *P. italicum* were stored at 20 °C for four days and at 15 °C for 12 days; while oranges inoculated with *P. digitatum* were stored at 15 °C for 15 days. Negative control samples were included for each trial. Control source fruits (whole and tissue sections) underwent the same steps as inoculated samples but were not inoculated with a fungal spore suspension.

### Disease incidence and severity measurements

After contact inoculation, disease incidence, and severity were measured daily for tomatoes and every two days for apples and oranges. Disease incidence was calculated as the percentage of fruits displaying visual signs of tissue maceration or soft rot. Disease severity was obtained by measuring lesion area (in mm^2^) from pictures taken at each time point using a Nikon D5100 DSLR Camera with 18–55 mm f/3.5–5.6 and a custom-made macro in the ImageJ software [24].

### Multispectral imaging for early lesion detection

High-resolution multispectral images were taken using a VideometerLab 4 (Videometer A/S, Herlev, Denmark) and processed with the VideometerLab software version 3.22.29. This equipment includes a sphere that uses strobe light-emitting diode (LED) technology to provide uniform and diffuse illumination. Reflectance images were taken at 19 wavelengths (365, 405, 430, 450, 470, 490, 515, 540, 570, 590, 630, 645, 660, 690, 780, 850, 880, 940, and 970 nm), including the longpass filters for a total of 50 spectral bands of the electromagnetic spectrum. Multispectral images for all target fruits were taken with the stem end pointed to the side and the equator in the center before and after contact-based inoculation.

For image analysis, pixels representing healthy and infected tissues were collected from a subset of fruit images from the total oranges. A normalized canonical discriminant analysis (nCDA) transformation based on the reflectance of each pixel was created to minimize the distance within classes and to maximize the distance among classes. A region of interest (ROI) was obtained from all images by applying a mask to segment the fruits from the background. All fruits were collected in a blob database, and the healthy and infected areas were extracted based on the previously created nCDA transformation. Shape and spectral features were extracted from individual blobs, including area and tristimulus components of color, such as hue and saturation. The SpectralMean feature extracts the reflectance mean of each fruit for the 50 spectral bands. Region MSI_Mean calculates a trimmed mean of transformed pixel values within the blob (every single fruit), and MSIThreshold measures the percentage or area of the blob region with a transformation value higher than the threshold, based on the nCDA model (derived from all the classes).

## Results

### Reproducibility assessment of the contact inoculation protocol on different pathosystems

**Fig. 2:**
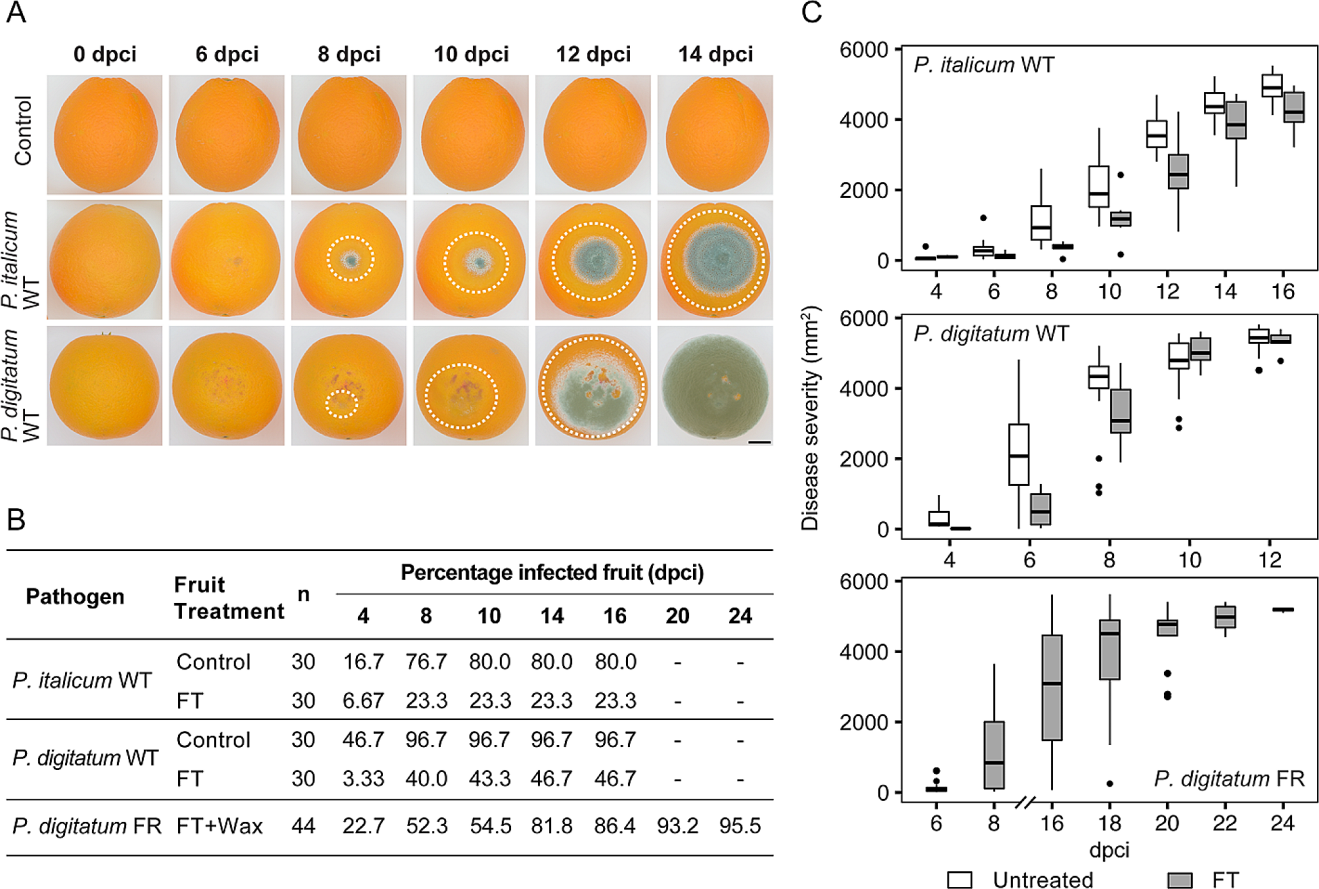
Oranges inoculated with *P. italicum* and *P. digitatum* using a non-wounding contact-based method. **(A)** Disease progression of infected oranges with *P. italicum* and *P. digitatum* wild-type compared to negative control (i.e., healthy source fruits in contact with a healthy target fruits) at selected time points between 0 and 14 days post-contact inoculation (dpci). White dotted lines highlight approximate lesion boundaries. Scale bar corresponds to 15 mm. **(B)** Disease incidence table of calculated percentage of infected fruits across evaluated dpci. **(C)** Disease severity box plots of lesion area progression on each fruit measured every other day. Gray-filled box plots represent fungicide-treated oranges, while white-filled box plots untreated oranges. WT: wild-type; FR: fungicide resistant; FT: fungicide treated

**Fig. 3:**
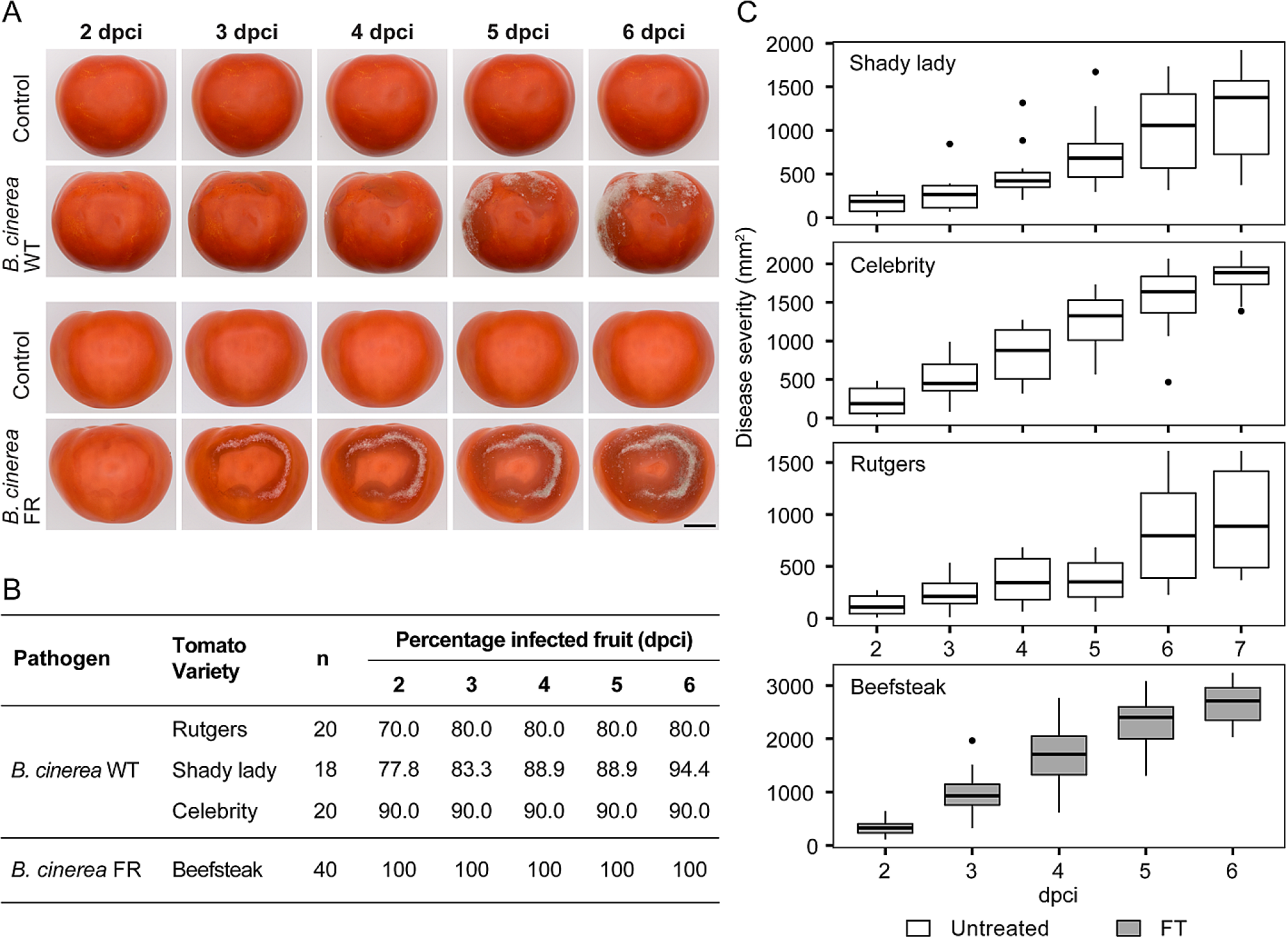
Tomatoes inoculated with *B. cinerea* using a non-wounding contact-based method. **(A)** Disease progression of contact-inoculated ‘Shady Lady’ tomatoes with *B. cinerea* wild-type and fungicide-resistant isolates compared to negative control (i.e., healthy source fruit tissues in contact with a healthy target fruit) at selected time points between 0 and 6 days post-contact inoculation (dpci). Scale bar corresponds to 20 mm. **(B)** Disease incidence table of calculated percentage of infected fruits across evaluated dpci. **(C)** Disease severity box plots of lesion area progression on each fruit measured daily. WT: wild-type; FR: fungicide resistant

**Fig. 4:**
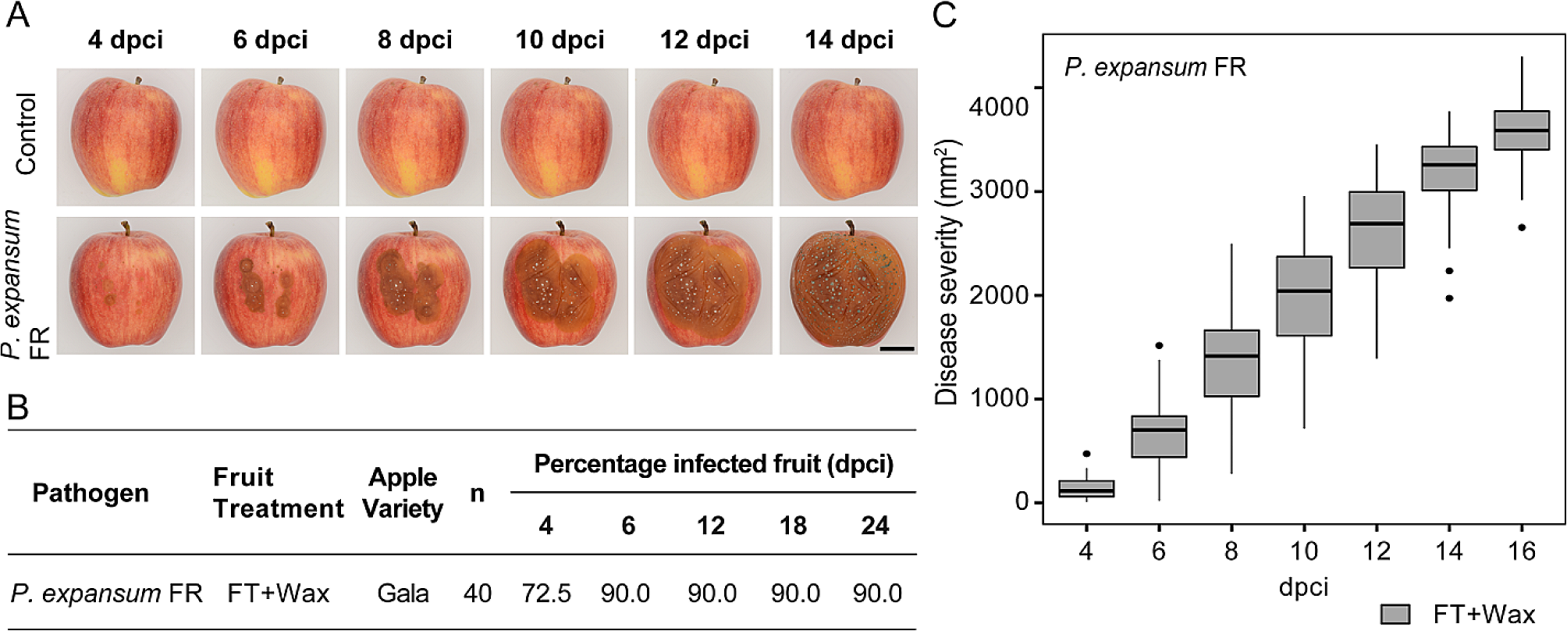
Apples inoculated with *P. expansum* using a non-wounding contact-based method. **(A)** Disease progression of infected apples with fungicide-resistant *P. expansum* compared to the negative control (i.e., healthy source fruit tissues in contact with a healthy target fruit) at selected time points between 0 and 14 days post-contact inoculation (dpci). Scale bar corresponds to 25 mm. **(B)** Disease incidence table of calculated percentage of infected fruits across evaluated dpci. **(C)** Disease severity box plots of lesion area progression on each fruit measured every other day. Gray-filled box plots represent data for fungicide and wax-treated apples. FR: fungicide resistant; FT: fungicide treated

After choosing the appropriate source inoculum type and incubation periods, we tested the reliability and reproducibility of the contact-based inoculation method using different fungal species and strains on target fruits with and without fungicide treatments (Table 1). In all the tests, the fungi spread from the contact points between the source and target fruit, causing tissue maceration and, in some cases, extensive mycelium growth (Figs. 2A and 3A, and 4A). No symptoms were evident when non-infected fruits or tissues were used as source inoculum to control for secondary or unintended infections.

Although we noticed some variation across different fruit commodities, fruit treatments, and fungal species and strains, most of our trials yielded disease incidence values of at least 80%, indicating the high performance of the contact-based inoculation method (Figs. 2B and 3B, and 4B). Additionally, we observed that established lesions in the target fruits continued to expand over time, confirming that the fungal pathogens tested in this study could colonize and complete their life cycle once they penetrated the fruits (Figs. 2C, 3C and 4C).

Wild-type (WT) strains (i.e., susceptible to fungicides) of *P. italicum* and *P. digitatum* reached a disease incidence of 80% and 96.7% after 10 and 8 dpci, respectively, in untreated target oranges. In contrast, when using fungicide-treated (FT) oranges, a low overall disease incidence was observed (from 23.3% to 46.7%) for *P. italicum* and *P. digitatum* WT strains after 8 dpci and 14 dpci, respectively (Fig. 2B). On the other hand, disease incidence in fungicide and wax treated oranges contact-inoculated with a *P. digitatum* fungicide-resistant (FR) strain was found to be 95.5%, which was similar to the untreated control oranges infected with *P. digitatum* WT (Fig. 2B). Lesion expansion in oranges varied depending on the target orange treatment and fungal isolate used (Fig. 2C). Overall, although lesion sizes were smaller for the FT oranges when using WT strains, the increase in lesion size across time points shows that the fungal pathogens tested in this study can colonize and complete their life cycle once they penetrate the target fruit.

Field-grown tomatoes of the ‘Celebrity’ hybrid cultivar showed a high disease incidence, reaching 90% across all evaluated time points when contact-inoculated with a *B. cinerea* WT strain (Fig. 3B). Meanwhile, other field-grown tomatoes from the ‘Rutgers’ variety and ‘Shady Lady’ hybrid cultivar achieved the maximum disease incidence at 4 dpci and 6 dpci, with 80% and 94.4%, respectively. Commercial, greenhouse-grown, hybrid ‘Beefsteak’ tomatoes showed 100% disease incidence at 2 dpci when contact-inoculated with a *B. cinerea* FR strain. Tomatoes that did not show any disease incidence remained uninfected throughout the trial. Similarly to oranges, lesion size development varied across tomato cultivars and strains used, but shows the fungal strains used can colonize the target fruits (Fig. 3C).

In apples, *P. expansum* FR infected 72.5% of the apples at 4 dpci and 90% at 6 dpci. The number of infected target apples remained unchanged until the last recorded time. Seven out of 40 apples never showed infection or lesion development from *P. expansum* and were considered as not infected during this study (Fig. 4B). For the infected apples, lesion sizes steadily increased throughout the duration of the evaluation period (Fig. 4C).

### Detecting early lesion development in oranges using multispectral imaging

**Fig. 5:**
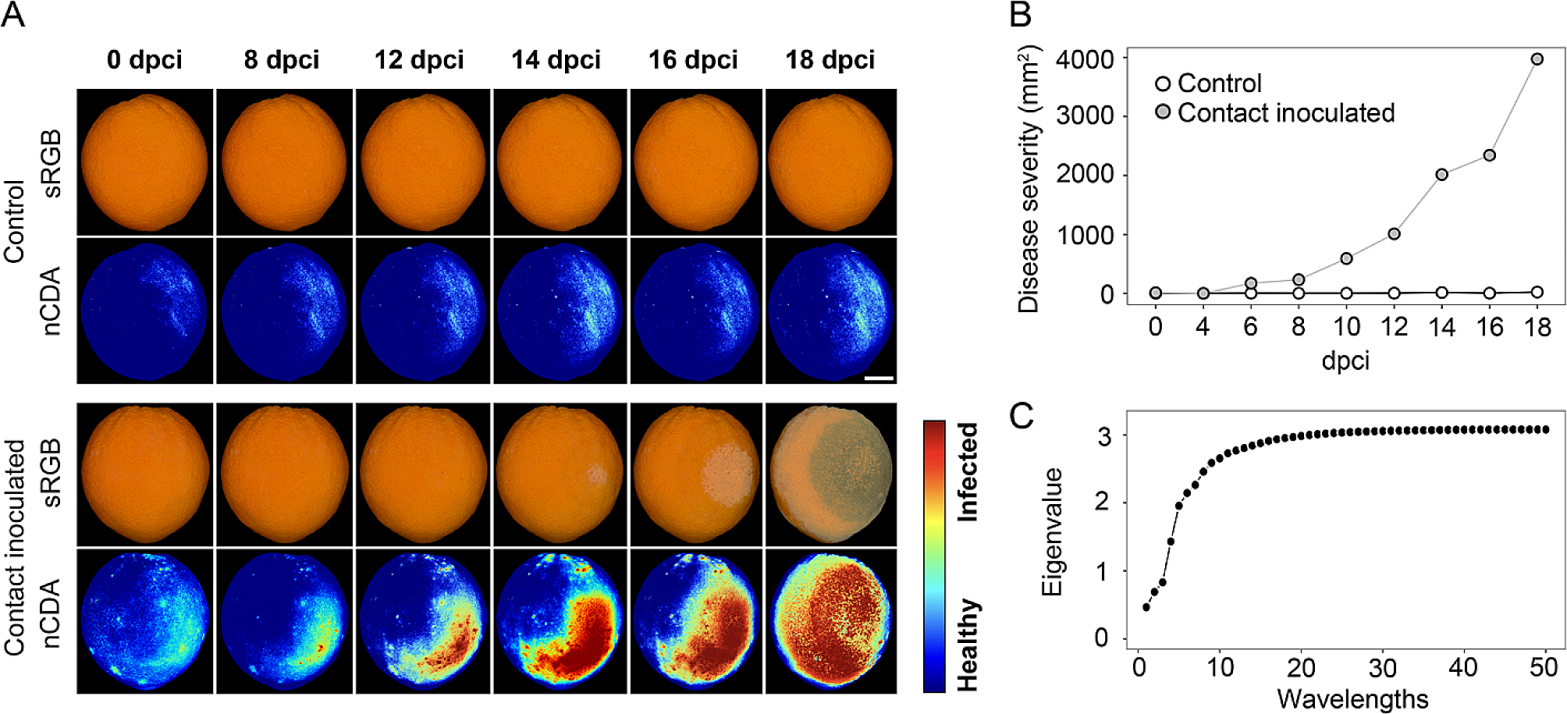
Visualization of disease progression in contact-inoculated oranges using multi-spectral imaging. **(A)** Comparison of negative control and contact-inoculated oranges with a fungicide-resistant strain of *P. digitatum* across different time points with raw (sRGB) and corresponding transformed images (nCDA). Color scale refers to individual transformed pixel values from healthy (blue or -2) to infected (red or 2). Scale bar corresponds to 15 mm. **(B)** Detection of infected tissue areas for both treatments across time. Transformed pixels above the standard threshold (green or 0). **(C)** Performance of the nCDA transformation (max. Eigenvalues) with an increasing number of combined wavelengths. All fruits used for this experiment were commercial fungicide- and wax-treated oranges. dpci: days post contact inoculation; nCDA: non-canonical discriminant analysis; sRGB: standard red, green, blue

In oranges, *Penicillium spp.* growth showed minimal visual progression and seemed limited to the contact point until 12 dpci (Fig. 2A). However, using multispectral imaging (MSI) we detected disease progression on the surface of target fruits, which was not apparent to the naked eye. Changes in the reflectance profiles of contact-inoculated oranges show that lesions appeared as early as 8 dpci and continued to expand even when mature lesions and visible mycelium were only obvious until 14 dpci and 18 dpci, respectively (Fig. 5A). On the other hand, practically no changes in the reflectance profile were observed for control fruits (i.e., healthy source fruits incubated in contact with a healthy target fruits). Furthermore, normalized canonical discriminant analysis (nCDA) transformation, which combines all wavelengths, could detect changes in areas where lesions were to be developed in earlier time points. These areas were calculated using a threshold (> 0) in the nCDA transformed scale ranging from healthy tissues (-2) to infected tissues (2) (Fig. 5A and B). The highest separation potential in the nCDA transformation (i.e., maximum eigenvalues) was obtained when combining about ten wavelengths with minor gain with additional wavelengths (Fig. 5C). Using MSI to monitor pathogen growth revealed the early onset of infection in oranges. It also can be used for sensitive quantification of lesion area before the disease is visible to the naked eye.

## Discussion

Consistent and reliable inoculation methods that mimic natural conditions and industry scenarios are key for the study of plant-pathogen interactions and the development of postharvest control methods. Here, we established a non-wounding, contact inoculation protocol that recreates the infections naturally occurring in the postharvest supply chain through nesting. We produced whole fruits or tissues that were infected and served as inoculum sources for spreading the disease to healthy fruit. In all fruits, except for fungicide-treated oranges contact inoculated with wild-type pathogen strains, disease incidence rates of 80% or higher were observed by the final evaluation time point, showing the effectiveness of the proposed methodology. Disease severity measurements were used to evaluate disease progression and growth behavior of fungal pathogens, confirming successful infections beyond the initial contact point in each fruit commodity. Even though disease incidence was lower in fungicide-treated oranges contact-inoculated with fungicide-sensitive pathogens, having successful infections in fungicide-treated oranges showed that the pathogens were capable of causing disease through this protocol, even in disadvantageous conditions. Furthermore, visualizing the fruits using MSI allowed us to confirm that, although lesions were not visible to the naked eye until 14 days post-contact inoculation (dpci) with *P. digitatum*, the fungus was established in the fruit tissue and growing by 8 dpci.

While successful *B. cinerea* infections were observed in all tomato trials, differences in disease incidence and severity values were observed across varieties. ‘Rutgers’ tomatoes exhibited greater variation between infection rates and a lower disease incidence than other tomato varieties. These differences could be due to surface characteristics, such as cuticle thickness and permeability, which influence the generation of pathogen-induced signals that activate defense responses [17, 25]. The ‘Rutgers’ tomatoes are more similar to processing types. Furthermore, thicker fruit cuticles provide higher resistance to initial *B. cinerea* infections [26, 27]. Also, differences in pathogen behavior were observed in this study, particularly between *Penicillium spp*. when contact inoculated in oranges and apples. *P. italicum* WT showed an earlier mycelium appearance as compared to *P. digitatum* WT, although it was the latter that covered the fruits completely in mycelium first. In apples, *P. expansum* showed a slower disease incidence and severity progression than the other commodities. This could be due to differences in infection mechanisms (e.g., host cell wall degrading enzymes, reactive oxygen species, toxins, among others) between fungal species [28, 29], as well as due to the fact that blue mold (*P. italicum* and *P. expansum*) develops better at cooler temperatures compared to green mold [12, 13].

For all three fruit commodities tested, a percentage of the fruits did not get infected, even after several weeks of monitoring. This could be because the fruit was able to halt pathogen infection at the contact point, preventing it from spreading throughout the tissues [27, 30, 31]. Another possibility is that, despite homogeneous incubation conditions, fruit-specific microclimates were not always conducive to disease development in all fruits, even though we ensured consistency of the technical aspects of the protocol.

The overall results of this study confirm that the non-wounding, contact-based inoculation method was effective in all fruit-pathogen interactions tested. Although an initial step of wound-inoculating the source fruit material is required, spread of the disease to healthy target fruits is done solely through contact between the tissues, and the target fruits remain unwounded throughout the entire procedure. This method holds promise for further application in other pathosystems by focusing on several key aspects. First, it is crucial to recognize the importance of the homogeneity of the fruits used as source inoculum and the initial 24 h during contact inoculation, as both play a significant role in the establishment and spread of fungal infections. For example, it is recommended that, if possible, fruits should come from the same location and supplier, and transportation-storage conditions should remain constant. Also, the incubation should be done at high humidity with some level of gas exchange (e.g., oxygen and CO_2_ diffusion), especially after the first day of contact. Second, investigating the contact time between source and target fruits is essential to ensure the accuracy and reproducibility of the inoculation method. Third, exploring the position of infected fruits or tissues, which serves as the source of inoculation, will help identify the most favorable conditions for efficient pathogen transfer between fruits through contact. Although we have not characterized the type of inoculum that is spreading from source to target fruit, our observations that source fruits or tissue sections without visible mycelium or sporulation were more effective in contact inoculation, we hypothesize that the disease is spread by fungal hyphae that are moving from infected tissues to healthy ones in search of nutrients. Ultimately, this protocol offers an effective and robust method for studying fruit-pathogen interactions and can be used to test the efficacy of postharvest treatments against persistent postharvest pathogens.

## Conclusion

This research is the first to develop an effective non-wounding, contact-based inoculation method that mimics the nesting phenomenon in postharvest conditions. The development of the method, from producing infected source fruits followed by setting the infected tissues in contact with the target fruits, simulates how infections occur during postharvest storage. The method was tested with various pathogens and fruit commodities, demonstrating its versatility, consistency, and applicability with a wide range of fruit-pathogen combinations.

## Data Availability

The datasets used and/or analyzed during the current study are available from the corresponding author on reasonable request.

## Abbreviations

RR: Red Ripe
dpa: days post-anthesis
TBZ: Thiabendazole
IMZ: Imazalil
WT: Wild-type
FR: Fungicide resistant
FT: Fungicide treated
dpci: days post-contact inoculation
RH: Relative humidity
LED: Light-emitting diode
nCDA: normalized canonical discriminant analysis
ROI: Region of interest
MSI: Multispectral images
sRGB: standard red, green, blue
